# Patient-Reported Opioid Consumption and Pain Intensity After Common Orthopedic and Urologic Surgical Procedures With Use of an Automated Text Messaging System

**DOI:** 10.1001/jamanetworkopen.2021.3243

**Published:** 2021-03-25

**Authors:** Anish K. Agarwal, Daniel Lee, Zarina Ali, Brian Sennett, Ruiying Xiong, Jessica Hemmons, Evan Spencer, Dina Abdel-Rahman, Rachel Kleinman, Hannah Lacko, Annamarie Horan, Mary Dooley, Eric Hume, Samir Mehta, M. Kit Delgado

**Affiliations:** 1 Department of Emergency Medicine, University of Pennsylvania, Philadelphia; 2 Leonard Davis Institute of Health Economics, University of Pennsylvania, Philadelphia; 3 Penn Medicine Center for Health Care Innovation, University of Pennsylvania, Philadelphia; 4 Department of Surgery, Division of Urology, University of Pennsylvania, Philadelphia; 5 Department of Neurosurgery, University of Pennsylvania, Philadelphia; 6 Department of Orthopaedic Surgery, University of Pennsylvania, Philadelphia; 7 Department of Biostatistics, Epidemiology, and Informatics, University of Pennsylvania, Philadelphia

## Abstract

**Importance:**

Surgeons must balance management of acute postoperative pain with opioid stewardship. Patient-centered methods that immediately evaluate pain and opioid consumption can be used to guide prescribing and shared decision-making.

**Objective:**

To assess the difference between the number of opioid tablets prescribed and the self-reported number of tablets taken as well as self-reported pain intensity and ability to manage pain after orthopedic and urologic procedures with use of an automated text messaging system.

**Design, Setting, and Participants:**

This quality improvement study was conducted at a large, urban academic health care system in Pennsylvania. Adult patients (aged ≥18 years) who underwent orthopedic and urologic procedures and received postoperative prescriptions for opioids were included. Data were collected prospectively using automated text messaging until postoperative day 28, from May 1 to December 31, 2019.

**Main Outcomes and Measures:**

The primary outcome was the difference between the number of opioid tablets prescribed and the patient-reported number of tablets taken (in oxycodone 5-mg tablet equivalents). Secondary outcomes were self-reported pain intensity (on a scale of 0-10, with 10 being the highest level of pain) and ability to manage pain (on a scale of 0-10, with 10 representing very able to control pain) after orthopedic and urologic procedures.

**Results:**

Of the 919 study participants, 742 (80.7%) underwent orthopedic procedures and 177 (19.2%) underwent urologic procedures. Among those who underwent orthopedic procedures, 384 (51.8%) were women, 491 (66.7%) were White, and the median age was 48 years (interquartile range [IQR], 32-61 years); 514 (69.8%) had an outpatient procedure. Among those who underwent urologic procedures, 145 (84.8%) were men, 138 (80.7%) were White, and the median age was 56 years (IQR, 40-67 years); 106 (62%) had an outpatient procedure. The mean (SD) pain score on day 4 after orthopedic procedures was 4.72 (2.54), with a mean (SD) change by day 21 of −0.40 (1.91). The mean (SD) ability to manage pain score on day 4 was 7.32 (2.59), with a mean (SD) change of −0.80 (2.72) by day 21. The mean (SD) pain score on day 4 after urologic procedures was 3.48 (2.43), with a mean (SD) change by day 21 of −1.50 (2.12). The mean (SD) ability to manage pain score on day 4 was 7.34 (2.81), with a mean (SD) change of 0.80 (1.75) by day 14. The median quantity of opioids prescribed for patients who underwent orthopedic procedures was high compared with self-reported consumption (20 tablets [IQR, 15-30 tablets] vs 6 tablets used [IQR, 0-14 tablets]), similar to findings for patients who underwent urologic procedures (7 tablets [IQR, 5-10 tablets] vs 1 tablet used [IQR, 0-4 tablets]). Over the study period, 9452 of 15 581 total tablets prescribed (60.7%) were unused. A total of 589 patients (64.1%) used less than half of the amount prescribed, and 256 patients (27.8%) did not use any opioids (179 [24.1%] who underwent orthopedic procedures and 77 [43.5%] who underwent urologic procedures).

**Conclusions and Relevance:**

In this quality improvement study of adult patients reporting use of opioids after common orthopedic and urologic surgical procedures through a text messaging system, the quantities of opioids prescribed and the quantity consumed differed. Patient-reported data collected through text messaging may support clinicians in tailoring prescriptions and guide shared decision-making to limit excess quantities of prescribed opioids.

## Key Points

**Question:**

Are the quantities of short-term opioid prescriptions after common orthopedic and urologic surgical procedures adequate or excessive as measured using automated text messaging?

**Findings:**

In this quality improvement study of 919 patients who underwent an orthopedic or urologic surgical procedure, most (61%) of the prescribed opioid tablets were reported as unused, and 28% of patients reported not using any opioids.

**Meaning:**

The findings suggest that opportunities exist to tailor opioid prescriptions and reduce excess quantities of opioids prescribed.

## Teaser

This quality improvement study used an automated test messaging system to assess the number of opioid tablets prescribed vs the self-reported number of tablets taken as well as self-reported pain intensity and ability to manage pain after orthopedic and urologic procedures.

## Introduction

Management of severe acute pain after surgery may require an opioid as part of an individual’s pain relief regimen^1,2^; however, clinicians must balance pain management approaches with the risks of opioid use within the context of state policies, which seek to mitigate these risks by limiting prescription of opioids for acute pain.^3,4^ The nonmedical use of prescription opioids remains a contributor to these risks and the second most common type of illicit drug use in the US.^5,6^ Up to 80% of opioids prescribed for acute pain after surgical procedures are left unused, and excessive quantities of opioids prescribed for acute pain have been associated with conversion to long-term use, overdose, and opioid use disorder.^7,8,9,10,11,12,13^ State policies have been criticized as a one-size-fits-all approach disconnected from the patient experience.^14,15^ Tailoring opioid prescribing to patients’ needs may be associated with reduced excess of prescribed opioids, but this practice remains challenging because patients’ experience of pain and responses to analgesia vary.^12,16,17,18^

A knowledge gap that has inhibited progress toward matching opioid prescriptions with anticipated patient needs is limited procedure-specific data to guide practitioners. Phone-based survey studies have started to fill this gap but have primarily focused on general surgical procedures and are labor intensive to replicate across the entire spectrum of surgical procedures.^12,13^ Despite wide national variability in opioid prescribing for acute pain after orthopedic and urologic surgical procedures, studies documenting patient opioid consumption after these high-volume procedures are limited.^19,20^ A learning health system approach^21,22^ would inform clinical practice with pragmatic patient-reported data and move toward a system that continually monitors use and informs prescribing. The rapid adoption of digital media (eg, text messaging or mobile surveys) has created scalable means of engaging patients and collecting real-time health data.^23,24,25^ Use of this approach for patient-reported pain and opioid use remains understudied.

In this study, we used an automated text messaging system to prospectively collect patient-reported data on pain intensity, ability to manage pain, and use of opioid prescriptions for acute pain after orthopedic and urologic surgical procedures as part of routine care within an academic health system. We also assessed the variations in opioid tablets prescribed and taken and patient pain outcomes over time after the procedures. The text messaging system was designed to develop a scalable system-level method of engaging patients to understand observable outcomes including pain intensity and opioid use in the postoperative period to improve future opioid prescribing.

## Methods

### Study Population and Period

All patients undergoing orthopedic and urologic surgical procedures at the Hospital of the University of Pennsylvania and Penn Presbyterian Medical Center were invited to participate in this quality improvement study. Recruitment of patients undergoing orthopedic procedures started on May 1, 2019, and recruitment of patients undergoing urologic procedures started in August 2019; patients who provided written informed consent through December 31, 2019, were included in this analysis. Patients were considered eligible if they were 18 years or older, were prescribed an opioid for postoperative control of acute pain, and had a verified mobile telephone number listed in the electronic health record. This study was approved by the institutional review board of the University of Pennsylvania. The study followed the Standards for Quality Improvement Reporting Excellence (SQUIRE) reporting guideline.^26^

### Data Collection

Eligible patients were identified from automated daily operative reports of all patients undergoing procedures by the surgeons in the eligible departments. The telephone number of each eligible patient was then entered into an automated text messaging platform (Mosio), which was programmed to prospectively collect data from patients on patient-reported outcomes and opioid consumption in the initial 28 days after an orthopedic or urologic procedure (eAppendix in the Supplement). The initial text message obtained electronic written consent to receive text messages and collect self-reported pain scores and use of opioid prescriptions. This process of electronic written consent via text messaging was approved by the University of Pennsylvania Health System’s privacy and safety committee and the University of Pennsylvania institutional review board. Patients had the ability to opt out at any time.

After patients provided consent, they were asked to self-report pain intensity on a scale of 0 to 10, with 10 being the highest level of pain; their ability to manage pain on a scale of 0 to 10, with 10 representing very able to control pain; and their prescription opioid use in number of tablets . Patients were then asked whether they planned to take opioids to manage pain in the upcoming days. Patients reporting current or planned future opioid use were queried on subsequent days 7, 14, 21, and 28. When a patient reported no use or no further use, text messaging ended. Prescribers were not directed to change their standard of care when discussing pain management, use of analgesics, or prescribing practice. In addition, text message questions were not framed to provide clinical guidance on managing pain but rather as means to collect patient-reported scores of pain intensity, ability to manage pain, and use of medication. Patient information was deidentified and stored securely. Demographic information, comorbidities, mental health history, and type of surgical procedure were obtained from the electronic health record.

### Statistical Analysis

The primary outcome of interest was the difference between the number of opioid tablets prescribed and the number of tablets taken. R, version 3.60 (R Project for Statistical Computing) was used for statistical analysis. A 2-sided α of .025 was considered statistically significant. All opioids were converted to equivalent doses of 5-mg oxycodone tablets.^27^ Primary orthopedic and urologic procedures were grouped by procedure type and anatomic region. Descriptive statistics were used to summarize patient demographic characteristics. Comparisons were done with the Fisher exact and χ^2^ tests for categorical variables and Kruskal-Wallis and Wilcoxon rank-sum tests for continuous variables. Patients who indicated not taking any opioids on day 4 were classified as using no opioids. Total number of pills consumed was calculated as the cumulative total of pills taken. Opioid pills prescribed and taken, pain intensity, and patients’ ability to manage pain are reported as median and interquartile range (IQR).

## Results

### Text Messaging and Recruitment

During the study period, 2037 patients were eligible to participate and were sent a text message invitation. A total of 919 (45.1%) provided written, electronic, informed consent through the automated system; 742 (80.7%) patients underwent orthopedic procedures (384 women [51.8%]), and 177 (19.2%) underwent urologic procedures (145 men [84.8%]) (eAppendix in the Supplement). Compared with nonconsenting patients, consenting patients who underwent orthopedic procedures were younger (median age, 48 [IQR, 32-61 years] vs 57 years [IQR, 39-67 years]; *P* < .001), were more likely to be White (491 [66.7%] vs 470 [54.2%]; *P* < .001), were more likely to have had an outpatient procedure (514 [69.8%] vs 460 [53.1%]; *P* < .001), had fewer comorbidities (Elixhauser Comorbidity Index score >4, 54 [7.3%] vs 129 [14.9%]; *P* < .001), and were more likely to be opioid naive, as defined by no record of an opioid prescription in the electronic health record over the past year (568 [77.2%] vs 611 [70.5%]; *P* = .003). Among the 177 patients who underwent urologic procedures, the median age was 56 years (IQR, 40-67 years), 138 (80.7%) were White, and 106 (62%) had an outpatient procedure. No statistically significant differences in age, sex, comorbidities, or mental health illnesses were observed between consenting and nonconsenting patients who underwent a urologic procedure (Table 1).

**Table 1.** Characteristics of Patients Consenting or Declining to Text Message Data Collection

Characteristic	Patients who underwent orthopedic surgical procedures			Patients who underwent urologic surgical procedures		
	Consented (n = 742)	No response or declined (n = 884)	*P* value	Consented (n = 177)	No response or declined (n = 234)	*P* value
Age, median (IQR)	48 (32-61)	57 (39-67)	<.001	56 (40-67)	60 (41-69)	.18
Sex, No. (%)						
Female	384 (51.8)	469 (53.1)	.80	32 (18.1)	59 (25.2)	.23
Male	358 (48.2)	415 (46.9)		145 (81.9)	175 (74.9)	
Race, No. (%)						
Asian	28 (3.8)	32 (3.7)	<.001	1 (0.6)	2 (0.9)	.16
Black	174 (23.6)	318 (36.7)		25 (14.6)	52 (23.6)	
Other or unknown	43 (5.8)	47 (5.4)		7 (4.1)	8 (3.6)	
White	491 (66.7)	470 (54.2)		138 (80.7)	158 (71.8)	
Hispanic, No. (%)						
Hispanic Latino or Black	15 (2.0)	8 (0.9)	.17	3 (1.8)	1 (0.5)	.42
Hispanic Latino or White	14 (1.9)	18 (2.1)		3 (1.8)	5 (2.3)	
Outpatient procedure, No. (%)	514 (69.8)	460 (53.1)	<.001	106 (62.0)	143 (65.0)	.61
History of anxiety or mood disorder, No. (%)	110 (14.9)	144 (16.6)	.40	25 (14.6)	27 (12.3)	.60
Elixhauser Comorbidity Index score, median (IQR)	1 (0-2)	1 (0-3)	<.001	1 (0-3)	1.5 (0-4)	.04
Long-term opioid use, No. (%)^a^	6 (0.8)	20 (2.3)	.03	1 (0.6)	4 (1.8)	.53
Opioid naive, No. (%)^b^	568 (77.2)	611 (70.5)	.003	129 (75.4)	153 (69.5)	.24
Opioid use disorder, No. (%)	12 (1.6)	31 (3.6)	.02	0 (0.0)	5 (2.3)	.13
Alcohol use disorder, No. (%)	6 (0.8)	18 (2.1)	.06	1 (0.6)	2 (0.9)	>.99
Tobacco use, No. (%)	14 (1.9)	42 (4.8)	.002	4 (2.3)	9 (4.1)	.50
NSAID prescribed, No. (%)	422 (56.9)	471 (53.3)	.16	36 (20.3)	56 (23.9)	.46

Abbreviations: IQR, interquartile range; NSAID, nonsteroidal anti-inflammatory.

^a^ Electronic health record evidence of 90 or more days of opioid prescriptions within the past 180 days.

^b^ No electronic health record evidence of an opioid prescription within 1 year before the index prescription.

### Pain Intensity and Ability to Manage Pain

Among participants who underwent orthopedic procedures, mean (SD) pain intensity on postoperative day 4 was 4.72 (2.54), with a mean (SD) change by 21 days after surgery of −0.40 (1.91) (Table 2). Mean (SD) self-reported ability to manage pain was high on day 4 (7.32 [2.59]) and remained high throughout the study period, with a mean (SD) change by day 21 of −0.80 (2.72) (Table 2). A similar pain trajectory was seen for patients who underwent urologic procedures (mean [SD] pain score on postoperative day 4, 3.48 [2.43]; mean [SD] change by day 21, −1.50 [2.12]). (Table 3). Overall, patients who underwent minor urologic procedures, including cystoscopy and scrotal procedures, did not report any pain 2 weeks after the procedure, and patients who underwent major open procedures reported low pain scores. The mean (SD) ability to manage pain score was high on day 4 (7.34 [2.81]) and improved over time (mean [SD] change at day 14, 0.80 [1.75]) (Table 3).

**Table 2.** Patient-Reported Pain Intensity and Ability to Manage Pain After Orthopedic Surgical Procedures^a^

Procedure type	No.	Initial pain score, day 4, mean (SD)	Change in pain score from day 4, mean (SD)^b^		
			Day 7	Day 14	Day 21
Ankle or pilon fractures	20	5.21 (2.92)	−1.50 (1.05)	1.00 (NA)	NA
Articular fractures around the knee	15	5.29 (2.20)	−1.00 (2.14)	−2.00 (0)	−2.00 (NA)
Carpal tunnel repair	19	4.06 (2.61)	−1.20 (1.79)	NA	NA
Complex knee arthroscopy	49	4.39 (2.48)	−1.12 (1.50)	−2.60 (1.52)	−2.00 (NA)
Hand fractures and dislocations	11	4.27 (2.05)	−2.00 (1.00)	−2.00 (NA)	6.00 (NA)
Hip arthroplasty	68	5.00 (2.15)	−1.33 (1.81)	−0.58 (2.09)	−0.50 (1.64)
Hip arthroscopy	24	4.00 (2.57)	0.50 (2.74)	−2.50 (4.95)	NA
I&D, removal of foreign body, implant, and other	26	5.72 (2.51)	−1.20 (1.55)	−0.67 (0.58)	−3.00 (4.24)
Knee arthroplasty	118	5.59 (2.19)	−0.64 (1.53)	−1.03 (1.50)	0 (1.36)
Long bone lower extremity fractures	13	5.85 (2.48)	−0.50 (2.35)	0 (1.41)	1.00 (NA)
Nondistal radius upper extremity fractures	22	6.38 (2.33)	−1.80 (1.30)	−1.50 (2.12)	NA
Shoulder arthroplasty	11	6.09 (1.97)	−1.67 (1.51)	−1.00 (3.61)	−1.00 (NA)
Shoulder arthroscopy	82	5.28 (2.44)	−0.83 (1.75)	−2.11 (1.69)	−1.25 (0.50)
Simple (minimally invasive) knee arthroscopy	188	3.72 (2.39)	−0.74 (1.45)	−1.91 (2.98)	NA
Upper extremity repair	46	3.60 (2.71)	−0.17 (1.83)	−2.00 (3.00)	NA
Overall	712	4.72 (2.54)	−0.89 (1.64)	−1.32 (2.04)	−0.40 (1.91)
	**No.**	**Initial ability to manage pain score, mean (SD)**	**Change in ability to manage pain from day 4, mean (SD)**		
Ankle or pilon fractures	20	7.21 (2.82)	−0.67 (1.86)	NA	NA
Articular fractures around the knee	15	7.71 (1.90)	−0.86 (1.86)	4.00 (0)	1.00 (NA)
Carpal tunnel repair	19	7.88 (2.57)	−0.80 (1.64)	NA	NA
Complex knee arthroscopy	49	7.83 (2.42)	−0.56 (2.10)	0.40 (1.14)	NA
Hand fractures and dislocations	11	7.73 (2.20)	0 (0)	NA	−2.00 (NA)
Hip arthroplasty	68	6.77 (2.78)	−0.45 (2.97)	0.79 (2.30)	−0.83 (1.17)
Hip arthroscopy	24	7.67 (2.81)	−1.17 (2.40)	4.00 (1.41)	NA
I&D, removal of foreign body, implant, and other	26	6.76 (2.70)	0.44 (2.13)	−1.00 (1.00)	0.50 (0.71)
Knee arthroplasty	118	6.94 (2.51)	−0.33 (2.58)	0.34 (2.79)	−0.67 (3.02)
Long bone lower extremity fractures	13	6.92 (2.19)	0.80 (0.45)	1.00 (0)	−1.00 (NA)
Nondistal radius upper extremity fractures	22	7.35 (2.16)	0.80 (1.64)	−2.00 (2.83)	NA
Shoulder arthroplasty	11	5.64 (2.98)	1.33 (1.21)	−3.00 (4.24)	NA
Shoulder arthroscopy	82	7.30 (2.32)	−0.36 (2.48)	−1.00 (4.58)	0.50 (2.38)
Simple (minimally invasive) knee arthroscopy	188	7.70 (2.68)	0.28 (3.32)	1.40 (1.90)	NA
Upper extremity repair	46	6.93 (3.19)	−1.17 (2.14)	3.67 (3.06)	NA
Overall	712	7.32 (2.59)	−0.20 (2.56)	0.47 (2.96)	−0.80 (2.72)

Abbreviations: I&D, incision and drainage; NA, not applicable.

^a^ Pain intensity was reported on a scale of 0 to 10, with 10 being the highest level of pain. The ability to manage pain was reported on a scale of 0 to 10, with 10 representing very able to control pain.

^b^ Some SDs are shown as NA because of low response rate at the given times.

**Table 3.** Patient-Reported Pain Intensity and Ability to Manage Pain After Urologic Surgical Procedures^a^

Procedure type	No.	Initial pain score, day 4, mean (SD)	Change in pain score from day 4, mean (SD)^b^		
			Day 7	Day 14	Day 21
Cystoscopy, stent, TURBT, and bladder biopsy	15	2.93 (3.03)	1.00 (NA)	NA	NA
Laparoscopic or robotic nephrectomy	17	4.00 (2.18)	−1.38 (2.72)	−2.67 (2.08)	NA
Major open surgery (open flank and open major)	17	3.71 (2.42)	−0.75 (1.49)	−0.33 (0.58)	−3.00 (NA)
Robotic prostatectomy	28	3.00 (1.85)	−1.00 (4.94)	NA	NA
Scrotal or open minor procedure (hernia and hydrocele)	15	3.67 (2.50)	−2.00 (1.41)	NA	NA
Transurethral resection of the prostate	10	3.70 (2.75)	−1.00 (0.00)	NA	NA
Ureteroscopy or stone procedure	21	3.76 (2.81)	−1.20 (1.64)	−1.00 (0.82)	NA
Urethroplasty, vaginal, and reconstructive procedures	17	3.94 (2.88)	−1.40 (1.14)	NA	NA
Vasectomy	29	3.14 (2.13)	−2.33 (0.58)	NA	NA
Overall	169	3.48 (2.43)	−1.20 (2.38)	−1.18 (1.47)	−1.50 (2.12)
	**No.**	**Initial ability to manage pain score, mean (SD)**	**Change in ability to manage pain from day 4, mean (SD)**		
Cystoscopy, stent, TURBT, and bladder biopsy	16	5.71 (3.93)	8.00 (NA)	NA	NA
Laparoscopic or robotic nephrectomy	17	7.53 (2.83)	1.57 (4.28)	1.67 (2.89)	NA
Major open surgery (open flank and open major)	18	7.71 (2.23)	0.86 (0.90)	1.33 (1.15)	NA
Robotic prostatectomy	30	8.14 (2.41)	1.50 (2.07)	NA	NA
Scrotal or open minor procedure (hernia and hydrocele)	16	6.57 (3.13)	0.50 (0.71)	NA	NA
Transurethral resection of the prostate	10	8.57 (1.51)	−0.50 (0.71)	−1.00 (NA)	NA
Ureteroscopy or stone procedure	22	7.05 (2.60)	−0.60 (1.52)	0 (0)	NA
Urethroplasty, vaginal, and reconstructive procedures	20	5.44 (3.05)	−0.50 (2.38)	NA	NA
Vasectomy	30	8.39 (2.15)	3.00 (2.00)	NA	NA
Overall	162	7.34 (2.81)	1.03 (2.70)	0.80 (1.75)	NA

Abbreviations: NA, not applicable; TURBT, transurethral resection of bladder tumor.

^a^ Pain intensity was reported on a scale of 0 to 10, with 10 being the highest level of pain. The ability to manage pain was reported on a scale of 0 to 10, with 10 representing very able to control pain.

^b^ Some SDs are shown as NA because of low response rate at the given times.

### Use of Prescribed Opioid Analgesics

**Figure 1.**
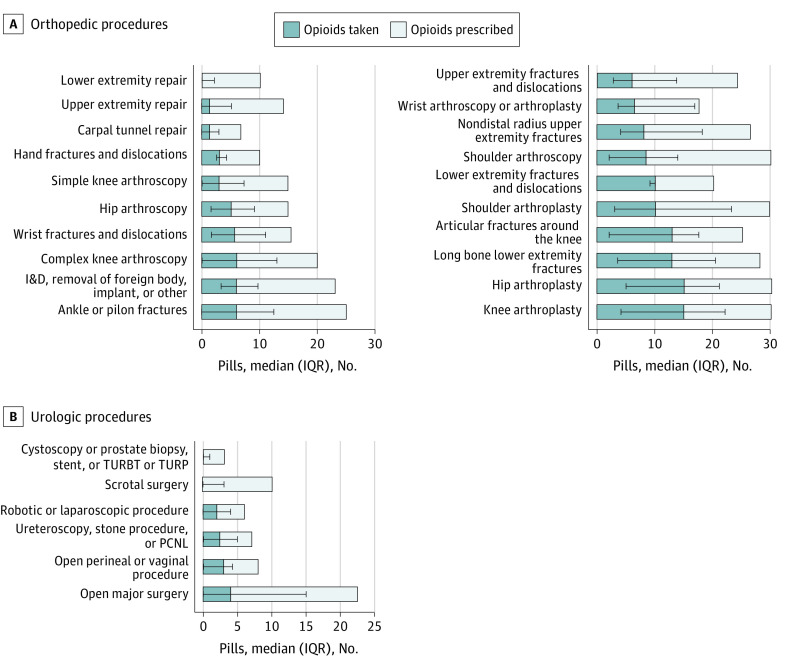
Opioid Prescribing and Patient-Reported Use I&D, incision and drainage; IQR, interquartile range; PCNL, percutaneous nephrostolithotomy; TURBT, transurethral resection of bladder tumor; TURP, transurethral resection of the prostate.

**Figure 2.**
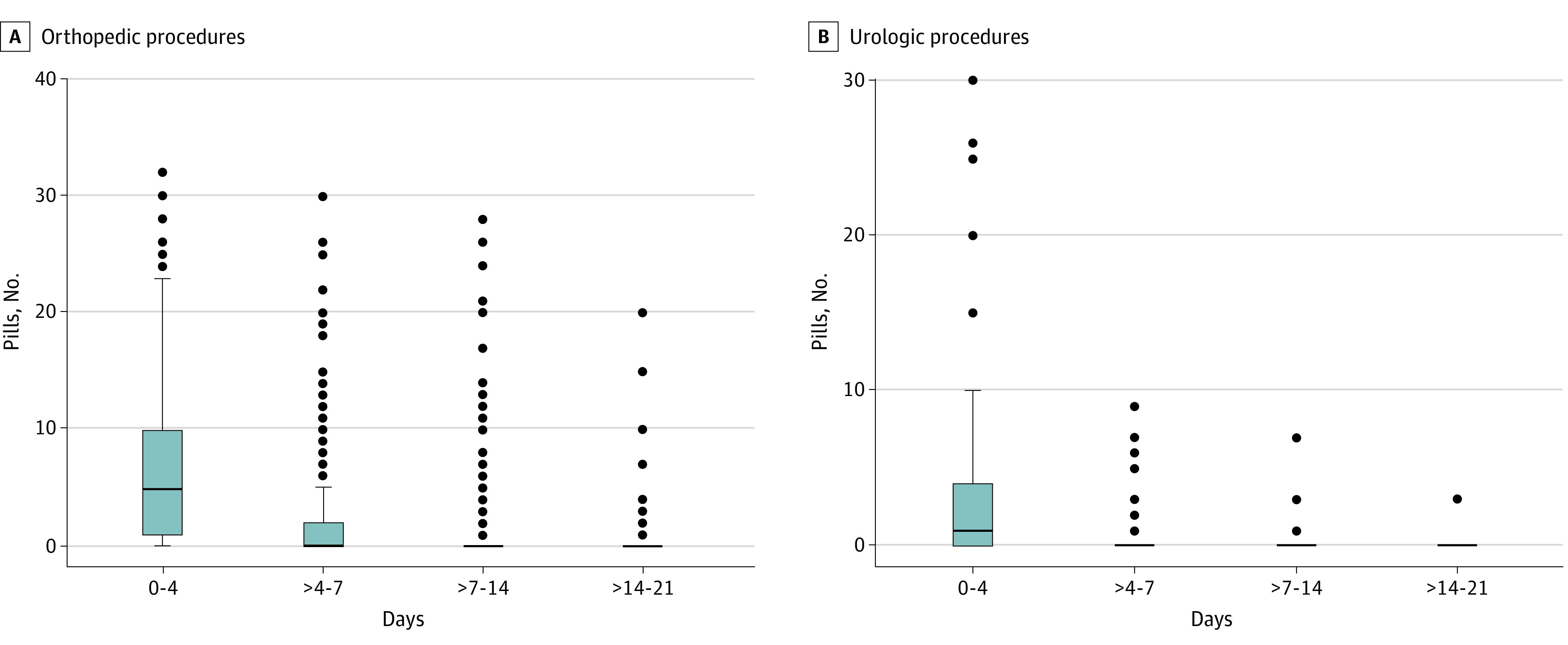
Patient-Reported Use of Opioid Tablets Within 21 Days of Surgery Horizonal lines indicate medians, and whiskers indicate interquartile ranges.

Consenting patients who underwent orthopedic procedures were prescribed a median number of 20 opioid tablets (IQR, 15-30 tablets), and 155 (21.1%) of these patients received an opioid refill within 30 days of surgery. Consenting patients who underwent urologic procedures were prescribed a median of 7 tablets (IQR, 5-10 tablets), and 19 (11.1%) received an opioid refill within 30 days of surgery. Prescription of nonsteroidal anti-inflammatory medication was similar for consenting patients and nonrespondents who underwent both types of procedures (Table 1). Variation existed in the quantity prescribed by procedure level (Figure 1A)., The median quantity of tablets prescribed for patients who underwent urologic procedures was 7 (IQR, 5-10), and the median quantity taken was 1 (IQR, 0-4) (Figure 1B). Similar variation for each procedure type was noted, with patients who had open abdominal procedures being prescribed the most and those who had cystoscopy, the least. Most tablets were taken within the first 4 postoperative days. The median quantity of tablets taken by patients who underwent orthopedic procedures was 5 (IQR, 0-14), with the greatest quantity taken within the first 4 days after hip arthroplasty (median, 15; IQR, 5-21). The median quantity taken within the first 4 days after all urologic procedures was 1 tablet (IQR, 0-4 tablets), with the highest being 4 tablets (IQR, 0-15 tablets) for open abdominal surgical procedures. By day 7, the median quantity of tablets taken for all procedures in this study was 0 (IQR, 0-2). Figure 2 shows opioid consumption over the study period.

### Total Amount of Opioids Prescribed

During the study period, 15 581 tablets were prescribed, and 9452 tablets (60.7%) were left unused. Of the total patient cohort, 589 (64.1%) used less than half of their prescribed tablets (464 [62.6%] who underwent orthopedic procedures and 125 [70.4%] who underwent urologic procedures). In total, 256 patients (27.8%) did not use any of the prescribed opioids (179 [24.1%] who underwent orthopedic procedures and 77 [43.5%] who underwent urologic procedures).

## Discussion

Promoting opioid stewardship for management of acute postoperative pain requires a nuanced and coordinated effort. In this study, we implemented a scalable automated text messaging system designed to immediately engage patients prescribed a short-term opioid treatment for relief of postoperative pain after orthopedic and urologic surgical procedures as part of routine care. Through this automated system, we sought to prospectively investigate opioid use and self-reported patient outcomes (eg, pain intensity and ability to manage pain). This study had 3 main findings. First, patient-centered data on acute pain and acute pain management could be observed and rapidly collected remotely through automated text messaging as part of routine care. Second, as in previous studies in which patient data were gathered after general surgical procedures,^13,28^ we found that for the entire spectrum of orthopedic and urologic procedures, 60.7% of opioid tablets were not taken after surgery, even in a health care system with relatively low baseline prescribing amounts. Third, the difference between short-term prescribing and patient-reported use emerged early and may provide additional context for prescribers in the development of patient-centered strategies to reduce excess quantities of prescription opioids while addressing acute, severe pain.

Previous retrospective research^9,10,11,12^ has described methods to query patients on their use of opioid prescriptions after surgery. These methods are time- and labor-intensive efforts requiring either telephone calls or paper surveys delivered to participants and returned via mail and may be limited by selection and recall bias as individuals attempt to remember their recovery experiences. Health care professionals have begun using digital methods to connect with patients.^23,24,25^ In prior studies, text messaging has been investigated as a means to monitor opioid use.^8^ This approach builds on prior research and incorporates the key concepts of a learning health system to serve as a continuous feedback mechanism to support clinical decision-making. This strategy has demonstrated the ability to rapidly capture patient-centered data on postoperative pain and pain management. This approach can be adapted for varying clinical settings and procedures and can gather patient-centered data prospectively and then use the data to inform clinical practices.

This study revealed that discordance among pain intensity, ability to manage pain, and opioid use emerged early in the postoperative recovery period. The mean (SD) pain score within the first week after an orthopedic procedure remained at 4.72 (2.54), with ability to manage pain being high. Low pain intensity was reported early after urologic procedures and improved quickly over a short period. Most opioid tablets were taken within the first 4 days after procedures, and patient reports of their ability to manage pain remained high during these times and subsequently as individuals took fewer or no opioid medications. Prior research has demonstrated similar discordance among pain intensity, ability to manage pain, and opioid use, but often, these data were collected 30 to 60 days after surgery and the analyses were retrospective in design and limited by recall. The approach described in this study provides a real-time and continuous method for clinicians to gain understanding of postoperative pain and opioid use and to build patient-informed and procedure-specific guidelines for future patients.

These results suggest key opportunities to address persistent gaps between opioid use for acute pain and prescribing, as 60.7% of tablets were left unused. Of patients who underwent orthopedic and urologic procedures, 24.1% and 43.5%, respectively, did not use any of the opioids prescribed to them. These patient-reported data may be used to guide prescribing. Median patient-reported use after all orthopedic procedures was 15 or fewer tablets (10 or fewer tablets used for 80% of procedures). For the patients who underwent urologic procedures, median reported used was 5 or fewer tablets. The potential to tailor prescribing of opioids to meet patients’ needs according to procedure and reduce excess prescribing remains high; national data reveal median prescribing to be 40 tablets for knee arthroscopy and 20 tablets for transurethral prostate or bladder tumor resections.^19,20^ In the study of orthopedic procedures, clinicians could proceed to develop procedure-specific and patient-informed prescribing guidelines to match use and need. As described in previous studies,^11,12,29,30^ aiming to meet the needs of the majority of patients remains a priority and challenge, whereby the 75th percentile of patient needs offers a beginning point for surgeons to tailor prescribing and reduce excess. For example, we found that a prescription for 7 tablets after simple knee arthroscopy would accommodate up to the 75th percentile of consenting patients’ reported use, as compared to the results, which showed a median of 15 tablets (IQR, 10-20 tablets) being prescribed . An additional approach could be used to guide broader specialty-level prescribing. In urology, with the exception of major open surgeries, we found that opioid prescriptions of 5 or fewer would address the 75th percentile of patient-reported use. Future research efforts should include study of the factors associated with the heterogeneity in patient-reported opioid use and how to ensure equity in meeting patient needs. Furthermore, these patient-reported data can be used to guide discussions between patients and prescribers toward development of shared decision models for prescribing opioids before surgery as they plan for postoperative pain management.

### Strengths and Limitations

The study has strengths. It applied a novel, low-tech method to engage patients and collect patient-reported data to inform clinical practice in a meaningful way. The study did not burden clinical staff with consenting and collecting data from patients. The approach was patient centered and could provide health care systems with a method of expanding the core concepts of a learning health system and continuous quality improvement.^31^

This study also has limitations. It was conducted within a single academic health care system with relatively low baseline prescribing compared with national prescribing patterns. Although incorporation of data collection via text messaging into routine care allowed us to solicit responses from all eligible patients, selection and nonresponder bias remained present because individuals had to opt in to the text messaging survey. Response rates were higher than those for standard patient experience surveys collected as part of routine care. Furthermore, participants needed to have regular access to a text message–capable device. Nonresponding patients were older and were more likely to be Black and to have comorbid conditions. Future efforts to develop guidelines using patient-reported data would benefit from assessment of heterogeneity in patient-reported pain needs in consideration of these characteristics to account for the degree of nonresponse in these populations. Nonetheless, to our knowledge, this study is among the first to prospectively engage and begin to investigate patient-reported outcomes in a remote and automated method immediately after high-volume, common surgical procedures when short-term opioids are prescribed. In addition, because we relied on self-reporting, patients might have altered their responses; however, no clinical changes were made to an individual’s care because participants were notified that data collection was only for research. The Hawthorne effect might have also contributed to the limitations because we asked patients about their pain and use of opioid medication early in the postoperative period during a period of increased media coverage and public awareness about the opioid crisis in the US.

## Conclusions

In this quality improvement study of adult patients reporting use of opioids after common orthopedic and urologic surgical procedures through a text messaging system, the quantities of opioids prescribed and the quantity consumed differed. Patient-reported data collected through text messaging may support clinicians in tailoring prescriptions and guide shared decision-making to limit excess quantities of prescribed opioids.

## Notes

Supplement. **eAppendix.**
