# Supplementary material for: Patient-Reported Opioid Consumption and Pain Intensity After Common Orthopedic and Urologic Surgical Procedures With Use of an Automated Text Messaging System
Source: JAMA Netw Open. 2021 Mar 25;4(3):e213243. doi: 10.1001/jamanetworkopen.2021.3243 (PMC7994954; doi:10.1001/jamanetworkopen.2021.3243)
Supplement: Supplement. — eAppendix. [file jamanetwopen-e213243-s001.pdf]

Supplementary Online Content

Agarwal AK, Lee D, Ali Z, et al. Patient-reported opioid consumption and pain intensity after common orthopedic and urologic surgical procedures with use of an automated text messaging system. *JAMA Netw Open*. 2021;4(3):e213243. doi:10.1001/jamanetworkopen.2021.3243

eAppendix.

This supplementary material has been provided by the authors to give readers additional information about their work.

© 2021 Agarwal AK et al. *JAMA Network Open*.

eAppendix

Text Messaging Script
Post-Operative Day 4

Consent: **Q1**

"Hi, this is Penn Medicine checking in about your recent procedure. Text 'YES' if we can ask a few questions about how you are managing your pain. Text 'STOP' anytime to opt-out. Click here: bit.ly/2HJCIg4 to learn more about this program.

Texting is not secure. Other people may be able to see information in text messages. By texting back 'YES' you are accepting this risk. Message & data rates may apply."

Q2:
We wanted to see how you are doing after your recent procedure. We may check in a few times.
[line break] How would you rate your pain over the past 24 hours? (from 0-10, 0 being no pain [please choose one number])."

LOGIC:

- If response ≠ 0-10 → REPLY *"I'm sorry. We don't understand. Please text back a number 0-10."*
- If no response in 3 hours → REPLY *"We missed your response. How would you rate your surgical pain over the past 24 hours? (from 0-10, 0 being no pain [please choose only one number]).*

Q3:
"We encourage you to contact your provider if your pain is too much to handle.
[line break] How have you been able to manage your pain since being home? From 0-10, 0 being not at all able [please choose one number]."

LOGIC:

- If response =0-10 → Proceed to **Q4**
- If response ≠ 0-10 → REPLY *"I'm sorry. We don't understand. Please text back a number between 0-10."*
- If no response in 3 hours → REPLY *"We missed your response. How have you been able to manage your pain since being home? From 0-10, 0 being not at all able [please choose one number]."*

Q4:
"We just have a few more questions. [line break] Have you taken any of the following medications for your pain since your procedure (Acetaminophen, Aspirin, Ibuprofen, or Naproxen)? [Y for yes or N for no]. "

LOGIC:

- If response = Y, N, yes, or no → Proceed to **Q5**
- If response ≠ Y, N, yes or no → REPLY *"I'm sorry. We don't understand. Please text back Y or N."*

Q5:
"Thanks, this information helps guide our practice. It looks like you were also prescribed [narcotic_RX] for your pain.
[line break]Have you taken this medication? [Y for yes or N for no]."

LOGIC:

- If response = Y or yes → Proceed to **Q6**
- If response = N or no → Proceed to **Q10**
- If response ≠ Y, N, yes or no → REPLY *"I'm sorry. We don't understand. Please text back Y or N."*

Q6: *"Can you estimate how many pills of [narcotic_RX] you have taken since your procedure? Please text back a number"*

© 2021 Agarwal AK et al. *JAMA Network Open*.

LOGIC:

- If response = Number→ Proceed to **Q7**
- If response ≠ Number→ REPLY *"I'm sorry. We don't understand. Please text back a number."*

Q7: *"Do you have any of these pills remaining? [Y/N]"*
LOGIC:

- If response = Y, yes → Proceed to **Q8**
- If response = N, no Proceed to **Q11**
- If response ≠ Y, N, yes or no → REPLY: *"I'm sorry. We don't understand. Please text back Y or N."*

Q8: *"Do you plan to continue taking [narcotic_RX] to control your pain? [Y/N]"*
LOGIC:

- If response = Y or yes → REPLY: *"Thanks so much for your feedback! We'll check back with you in a few days. If you have any questions or concerns about your care or health please contact your surgical team. Have a nice day!"**(Q9 ON MOSIO)***

---This patient will be texted in 1 week---

- If response = N or no → **Move to Q10**
- If response ≠ Y, N, yes or no → REPLY: *"I'm sorry. We don't understand. Please text back Y or N."*

Q10: "Have you disposed of your unused %narcotic% medication? [Y/N]
LOGIC:

- If response = Y or Yes → That's great to hear. It's important to dispose of your unused medication. Just one more question for you: [line break] *"How many days did it take for your pain to improve after your procedure? Please text back a number."* ***(Q12 ON MOSIO)***
- If response = N or No → It's important to dispose of your unused medication. Penn pharmacies will take them back. Find locations here: https://bit.ly/2VQPekA. [line break] Just one more question for you: *"How many days did it take for your pain to improve after your procedure? Please text back a number."* ***(Q13 ON MOSIO)***

Q11: *"Thank you so much! Just one more question for you. How many days did it take for your pain to improve after your procedure?"*
LOGIC:

- If response = Number → REPLY "Thanks so much for your feedback! If you have any questions or concerns about your care or health, please contact your surgical team. (**Q14 ON MOSIO)**

- If response ≠ Number→ REPLY *"I'm sorry. We don't understand. Please text back a number."*

---END MARK COMPLETE---

Post-Operative Days 7, 14, and 21

Q1:

© 2021 Agarwal AK et al. *JAMA Network Open*.

"Hi, this is Penny Bot from Penn Medicine! We're checking back in to see how you're doing. Text STOP anytime to opt out. [line break] How would you rate your pain over the past 24 hours? (from 0-10, 0 being no pain [please choose one number]).

LOGIC:

- If response ≠ 0-10 → REPLY *"I'm sorry. We don't understand. Please text back a number 0-10."*

Q2:
*"We encourage you to contact your provider if your pain is too much to handle. [line break] How have you been able to manage your pain over the past 3 days**("over the past week" if days 14 and 21)**? From 0-10, 0 being not at all able [please choose only one number]."*

LOGIC:

- If response =0-10 → Proceed to **Q3**
- If response ≠ 0-10 → REPLY *"I'm sorry. We don't understand. Please text back a number between 0-10."*

Q3:
*"We just have a few more questions.[line break] Have you taken any of the following medications for your pain over the past 3 days**("over the past week" if days 14 and 21)**(Acetaminophen, Aspirin, Ibuprofen, or Naproxen)? [Y for yes or N for no]. "*

LOGIC:

- If response = Y, N, yes, or no → Proceed to **Q4**
- If response ≠ Y, N, yes or no → REPLY *"I'm sorry. We don't understand. Please text back Y or N."*

Q4:
*"Thanks, this information helps guide our practice. Have you taken [narcotic_RX] for your pain over the past 3 days**("over the past week" if days 14 and 21)**? [Y for yes or N for no]."*

LOGIC:

- If response = Y or yes → Proceed to **Q5**
- If response = N or no → Proceed to **Q9**
- If response ≠ Y, N, yes or no → REPLY *"I'm sorry. We don't understand. Please text back Y or N."*

Q5: *"Can you estimate how many pills of [narcotic_RX] you have taken in the past 3 days? Please text back a number."*

LOGIC:

- If response = Number→ Proceed to **Q6**
- If response ≠ Number→ REPLY *"I'm sorry. We don't understand. Please text back a number."*

Q6: *"Do you have any of these pills remaining? [Y/N]"*
LOGIC:

- If response = Y, yes → Proceed to **Q7**
- If response = N, no → Proceed to **Q10**
- If response ≠ Y, N, yes or no → REPLY: *"I'm sorry. We don't understand. Please text back Y or N."*

Q7: *"Do you plan to continue taking [narcotic_RX] to control your pain? [Y/N]"*
LOGIC:

- If response = Y or yes → REPLY: *"Thanks so much for your feedback! We'll check back with you in a few days. If you have any questions or concerns about your care or health please contact your provider. Have a nice day!"* ***(Q8 ON MOSIO)***

© 2021 Agarwal AK et al. *JAMA Network Open*.

---This patient will be texted in 1 week---

- If response = N or no → **Move to Q9.**
- If response ≠ Y, N, yes or no → REPLY: *"I'm sorry. We don't understand. Please text back Y or N."*

Q9: "Have you disposed of your unused %narcotic% medication? [Y/N]
LOGIC:

- If response = Y or Yes → That's great to hear. It's important to dispose of your unused medication. Just one more question for you: [line break] *"How many days did it take for your pain to improve after your procedure? Please text back a number." **(Q11 ON MOSIO)***
- If response = N or No → It's important to dispose of your unused medication. Penn pharmacies will take them back. Find locations here: https://bit.ly/2VQPekA. [line break] Just one more question for you: *"How many days did it take for your pain to improve after your procedure? Please text back a number." **(Q12 ON MOSIO)***

Q10:
"Thank you so much! Just one more question for you. How many days did it take for your pain to improve after your procedure?"

LOGIC:

- If response = Number→ REPLY "Thanks so much for your feedback! If you have any questions or concerns about your care or health please contact your surgical team." (**Q13 ON MOSIO**)

- If response ≠ Number→ REPLY *"I'm sorry. We don't understand. Please text back a number."*

---END MARK COMPLETE---

Day 28

Q1:
"Hi, this is Penny Bot from Penn Medicine! We're checking back in to see how you're doing. [line break] How would you rate your surgical pain over the past 24 hours? (from 0-10, 0 being no pain [please choose one number]).

LOGIC:

- If response ≠ 0-10 → REPLY *"I'm sorry. We don't understand. Please text back a number 0-10."*

Q2:
"We encourage you to contact your surgical team if your pain is too much to handle. [line break] How have you been able to manage your pain over the past week? From 0-10, 0 being not at all able [please choose only one number]."

LOGIC:

- If response =0-10 → Proceed to **Q3**
- If response ≠ 0-10 → REPLY *"I'm sorry. We don't understand. Please text back a number between 0-10."*

Q3:
"We appreciate your response. We just have a few more questions.[line break] Have you taken any of the following medications for your pain over the past week(Acetaminophen, Aspirin, Ibuprofen, or Naproxen)? [Y for yes or N for no]. "

LOGIC:

© 2021 Agarwal AK et al. *JAMA Network Open*.

- If response = Y, N, yes, or no → Proceed to **Q4**
- If response ≠ Y, N, yes or no → REPLY *"I'm sorry. We don't understand. Please text back Y or N."*

Q4:
"Thanks, this information helps guide our practice. Have you taken [narcotic_RX] for your pain over the past week? [Y for yes or N for no]."

LOGIC:

- If response = Y or yes → Proceed to **Q5**
- If response = N or no → Proceed to **Q9**
- If response ≠ Y, N, yes or no → REPLY *"I'm sorry. We don't understand. Please text back Y or N."*

Q5: *"Can you estimate how many pills of [narcotic_RX] you have taken over the past week? Please text back a number"*
LOGIC:

- If response = Number→ Proceed to **Q6**
- If response ≠ Number→ REPLY *"I'm sorry. We don't understand. Please text back a number."*

Q6: *"Do you have any of these pills remaining? [Y/N]"*
LOGIC:

- If response = Y, yes → Proceed to **Q7**
- If response = N, no → Proceed to **Q9**
- If response ≠ Y, N, yes or no → REPLY: *"I'm sorry. We don't understand. Please text back Y or N."*

Q7: *"Do you plan to continue taking [narcotic_RX] to control your pain? [Y/N]"*
LOGIC:

- If response = Y or yes → **Proceed to Q10**

- If response = N or no → **Move to Q8.**
- If response ≠ Y, N, yes or no → REPLY: *"I'm sorry. We don't understand. Please text back Y or N."*

Q8: "*Have you disposed of your unused %narcotic% medication? [Y/N]*
LOGIC:

- If response = Y or Yes → That's great to hear. It's important to dispose of your unused medication. Just one more question for you: [line break] *"How many days did it take for your pain to improve after your procedure? Please text back a number."****(Q11)***
- If response = N or No → It's important to dispose of your unused medication. Penn pharmacies will take them back. Find locations here: https://bit.ly/2VQPekA. [line break] Just one more question for you: *"How many days did it take for your pain to improve after your procedure? Please text back a number."* ***(Q12)***

Q9:
"Thank you so much! Just one more question for you. How many days did it take for your pain to improve after your procedure?"

LOGIC:

- If response = Number→ REPLY "Thanks so much for your feedback! If you have any questions or concerns about your care or health please contact your surgical team." (**Q13**)

© 2021 Agarwal AK et al. *JAMA Network Open*.

- If response ≠ Number→ REPLY *"I'm sorry. We don't understand. Please text back a number."*

Q10:
Thanks! When you're finished, it's important to dispose of unused %narcotic%. Penn pharmacies will take them. Find locations here: https://bit.ly/2VQPekA. [line break] *Just one more question for you. How many days did it take for your pain to improve after your procedure?"*

LOGIC:

- If response = Number→ REPLY "Thanks so much for your feedback! If you have any questions or concerns about your care or health please contact your surgical team." (**Q14)**

- If response ≠ Number→ REPLY *"I'm sorry. We don't understand. Please text back a number."*

<u>Flowchart of respondents over time:</u>

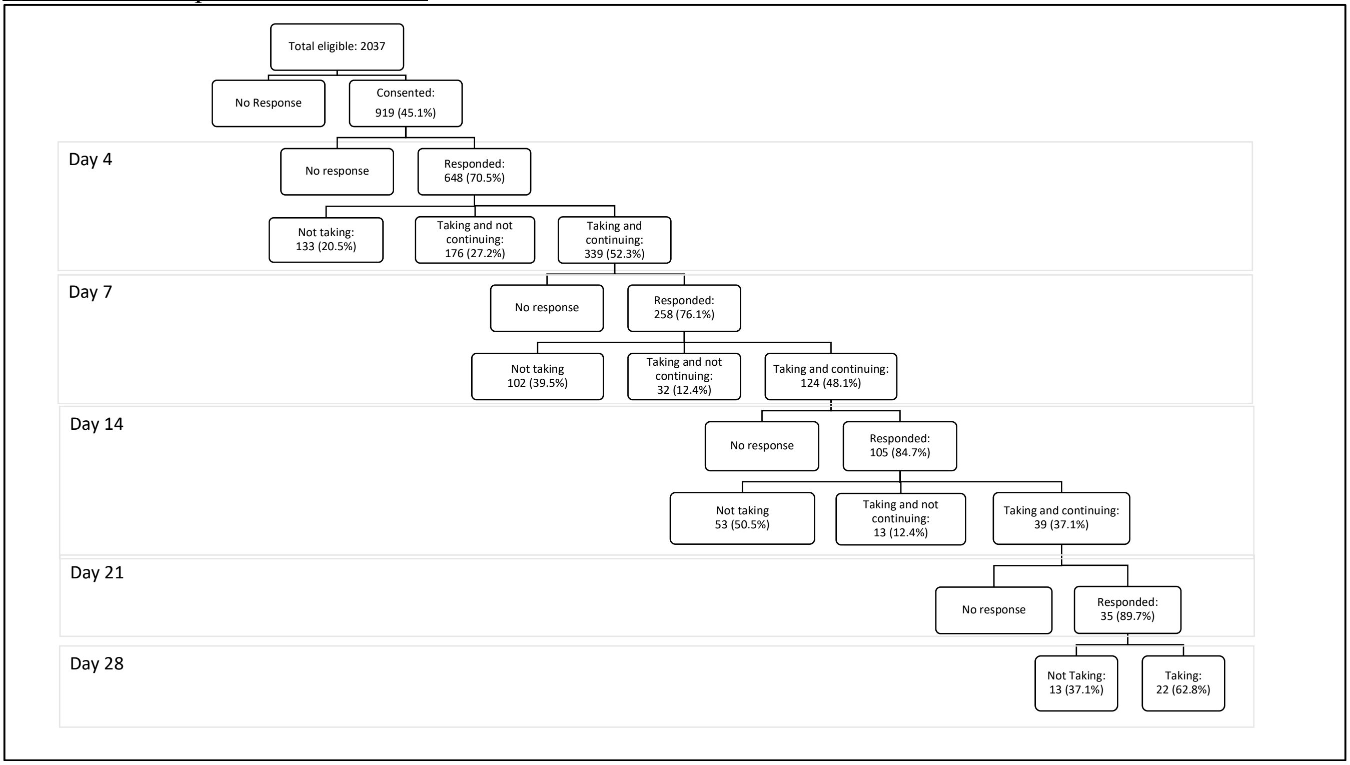

© 2021 Agarwal AK et al. *JAMA Network Open*.
